# Lactic Acidosis in Diabetic Population: Is Metformin Implicated? Results of a Matched Case-Control Study Performed on the Type 2 Diabetes Population of Grenoble Hospital University

**DOI:** 10.1155/2016/3545914

**Published:** 2016-02-29

**Authors:** Marion Lepelley, Joris Giai, Nassima Yahiaoui, Sébastien Chanoine, Céline Villier

**Affiliations:** ^1^Centre Régional de Pharmacovigilance, Pôle Santé Publique, Centre Hospitalier Universitaire de Grenoble, 38000 Grenoble, France; ^2^Service de Biostatistique des Hospices Civils de Lyon, 69002 Lyon, France; ^3^Laboratoire Biostatistique Santé, UCBL, Equipe de l'UMR CNRS 5558, 69495 Pierre-Bénite Cedex, France; ^4^Pôle Pharmacie, Centre Hospitalier Universitaire de Grenoble, 38000 Grenoble, France; ^5^Université Grenoble Alpes, 38000 Grenoble, France

## Abstract

*Introduction*. To evaluate the strength of association between lactic acidosis (LA) and well-recognized risk factors for LA, particularly the weight of metformin.* Methods*. This study is a matched case-control analysis concerning the type 2 diabetes population from Grenoble Hospital University. Cases of LA were defined biologically with pH < 7.35 and lactates > 5 mmol/L. They were matched to 2 controls defined as type 2 diabetic inpatients who did not present a LA during the study period. We performed a conditional logistic regression.* Results*. We included 302 cases and 604 controls; mean age was 69.5 years (SD 11.93). Intercurrent diseases were significantly associated with LA. Chronic medical conditions had a minor impact on LA incidence, except hepatocellular dysfunction. Metformin was significantly associated with a higher LA probability in case of acute kidney injury (AKI) (OR = 1.79; *p* value = 0.020) but not in patients without AKI.* Discussion and Conclusions*. According to this study, metformin, compared to acute medical conditions, seemed not to be associated with LA in patients with type 2 diabetes; however in case of AKI, metformin may be associated with LA.

## 1. Introduction

According to the World Health Organization, diabetic population should reach almost 600 million worldwide, in 2035 [1]. When medication is required, metformin is the first-line treatment in type 2 diabetes due to its superiority in reducing cardiovascular events and morbidity-mortality, compared to insulin and sulfonylureas [2–4].

The safety profile of metformin is well established: gastrointestinal disorders are the most frequent adverse effect. Lactic acidosis (LA) is a very rare but potentially severe adverse effect resulting from hepatic gluconeogenesis and mitochondrial respiration inhibition.

Causality between metformin and the occurrence of LA is a matter for debate and remains controversial. According to several studies, the risk of developing a LA due to metformin exposure is low, ranging from 2 to 9 cases per 100 000 patient-years [5–8]. A review of prospective trials and observational cohort studies did not find an increased risk of LA with metformin compared to other antihyperglycemic treatments [7]. A nested case-control analysis did not find any difference concerning occurrence of LA between metformin and sulfonylureas groups but identified risk factors for LA in all case subjects [8]. Furthermore, many cases of LA are reported in patients treated with metformin when presenting acute kidney failure due to dehydration or exposure to nephrotoxic drugs such as nonsteroidal anti-inflammatory drugs (NSAID), diuretics, and angiotensin converting enzyme (ACE) inhibitor. The spontaneous reporting of such cases is currently growing.

The mortality associated with this rare adverse drug effect is 50% with all biguanides [9]. Recently, it was estimated to be around 26 to 30% with metformin [5, 10]. When measured, plasmatic level of metformin is difficult to interpret; its correlation with lactate levels has not been demonstrated [7]. In case of LA, metformin level is significantly higher in survivors than in nonsurvivors [11].

This apparent discrepancy between clinical trials and real life probably results from the presence of uncontrolled intercurrent diseases.

Most contraindications, special warnings, and precautions for use labelled in the metformin summary product characteristics are aimed at minimizing this risk. Recently, contraindications have been judged unjustified and too restrictive [12]. For example, metformin is more and more prescribed on the basis of studies in moderate chronic renal failure or in case of cardiac failure [13–16]. It has been estimated that around half the patients would be deprived of this useful drug by a strict application of recommendations [13, 17, 18]. The potential consequence of such a change in prescription pattern is that intercurrent diseases could lead to more severe LA.

In this context, we conducted a study to evaluate the strength of association between the occurrence of LA in patients suffering type 2 diabetes and the well-recognized risk factors for LA and particularly to determine the impact of a metformin treatment among all these factors. These risk factors for LA were related to an underlying chronic condition or to an intercurrent acute disease.

## 2. Methods

We performed a matched case-control study on the type 2 diabetic population from Grenoble University Hospital, between 1 January 2008 and 31 December 2011.

### 2.1. Population, Cases, and Controls

The source population included every inpatient with type 2 diabetes admitted at Grenoble University Hospital between 2008 and 2011, according to the 10th Edition of the International Classification of Diseases (ICD). Considering potential coding approximations, we selected 3 ICD codes: non-insulin-dependent diabetes mellitus (E11), other specified diabetes mellitus (E13), and unspecified diabetes mellitus (E14). To exclude potential type 1 diabetic patients, we rejected patients under 30 years of age.

Every type 2 diabetic inpatient who presented a LA between 2008 and 2011 was considered as a potential case. According to Cohen and Wood's definition, LA was defined biologically as a metabolic acidosis characterized by an arterial pH < 7.35 and a lactate level > 5 mmol/L [19]. Group control included type 2 diabetic inpatients who did not present a LA during the study period. We decided to match one case to two controls, considering the rareness of controls with extreme ages. Our choice was to keep all cases included in the study.

Two controls were matched to every case based on gender, year of birth, and date of hospitalization. When more than two controls were available for matching, the choice was made at random. The case and its matched controls were excluded in case of missing data or ICD coding errors.

### 2.2. Potential Risk Factors and Exposure to Metformin

The list of LA risk factors was established based on the Cohen-Woods classification [19], on the analysis of case-reports in the literature and spontaneous reports of LA in the French national pharmacovigilance database. We distinguished chronic medical conditions, concomitant therapies, and intercurrent diseases.

Exposure to metformin and potential risk factors for LA were sought in every computerized medical record shortly before the date of diagnosis of LA. When necessary, we checked results of laboratory exams to be quite sure intercurrent diseases were present before pH and lactates became pathologic. Fatal outcome was also investigated for every patient.

Chronic medical conditions investigated were chronic kidney disease (CKD), hepatocellular dysfunction, chronic respiratory failure, chronic heart failure, neoplasia, mitochondrial dysfunction, and pyridoxine deficit.

Concomitant therapies investigated were ACE inhibitors, angiotensin II receptor antagonists (ARA), diuretics, antiretroviral (ARV) drugs, NSAID, metformin, insulin, and iodinated contrast media (CM).

Intercurrent diseases investigated were shock (cardiac, hypovolemic, and septic), severe anemia, dehydration (diarrhea, vomiting), acute kidney injury (AKI), acute hepatic failure, acute respiratory failure, myocardial infarction, acute decompensated heart failure, sepsis, convulsions, intense muscular effort, and acute artery occlusion.

### 2.3. Statistical Analysis

Statistical analyses were performed using STATA software (version 11.0, STATA Corp, College Station, Texas). Quantitative data were displayed as means and standard deviations (SD). Qualitative data were expressed with number of subjects and percentages. In case of weak effective, variables were excluded or grouped. The alpha risk was set at 5%.

#### 2.3.1. Univariate Analysis

We performed McNemar chi-squared tests and Student's tests for matched data. Covariates with low effectives (<5%) were excluded. Covariates were included for further analysis if *p* value < 0.25. Multicollinearity analysis of all covariates was performed.

#### 2.3.2. Multivariate Analysis

We used a model backward elimination process for the conditional logistic regression. After each step, a likelihood-ratio test was performed to compare models. Metformin was forced in the model despite its nonsignificance.

Interactions were searched in the model of conditional logistic regression. When an interaction was found, we achieved logistic regression with stratification.

A Hosmer-Lemeshow test was achieved for the final multivariate model. A model is considered suitable if the *p* value of this test exceeds 5%.

In order to assess the goodness of fit of the final model, we estimated its discriminatory power by using Receiver Operator Characteristic (ROC) curve and by calculating its sensitivity and specificity. Goodness of fit is considered to be acceptable if the area under the ROC curve is comprised between 0.7 and 0.8, good between 0.8 and 0.9, excellent above 0.9.

## 3. Results

### 3.1. Population Study

**Figure 1:**
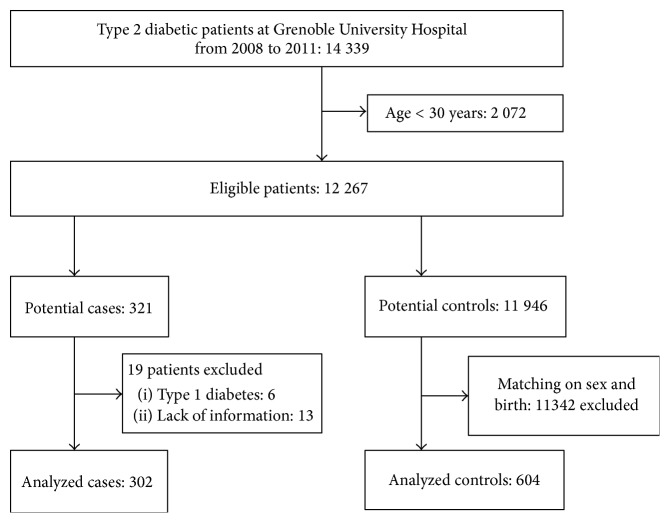
Study flow chart.

Initial list of type 2 diabetic patients contained a large number of patients, more than 14 000. After application of exclusion criteria, our source population included 12 267 eligible subjects. The flow chart of our study is presented in Figure 1.

### 3.2. Cases and Controls

The number of cases, that is, type 2 diabetic patients who presented a LA during 2008 and 2011, was 321. Nineteen cases were excluded because of coding errors (6 patients suffering type 1 diabetes) or lack of information (13 patients). Thus, a total of 302 cases were analyzed. They were matched to 604 controls. The final population study included 906 patients (Table 1).

There was no difference between cases and controls regarding age and sex, according to the study design; most chronic medical conditions: existence of CKD, mitochondrial dysfunction, neoplasia, or pyridoxine deficit; and most concomitant therapies: ACE inhibitors, ARA, diuretics, ARV drugs, and insulin.

Cases were significantly more affected by hepatic, cardiac, respiratory chronic diseases or advanced renal failure; more exposed to NSAID and iodinated CM; and more affected by any intercurrent disease. Mortality was logically higher among cases.

Controls were significantly more treated by metformin than cases.

### 3.3. Strength of Association between Covariates and Occurrence of LA

#### 3.3.1. Univariate Analysis

Due to weak effective (<5%), for further analyses we included the following covariates among chronic conditions: CKD, hepatocellular dysfunction, chronic respiratory failure, heart failure, and neoplasia; among concomitant therapies: ACE inhibitors, ARA, diuretics, metformin, insulin, and iodinated CM; and among intercurrent diseases: AKI, acute respiratory failure, acute heart failure, and sepsis. We defined 2 groups of CKD (instead of 4), namely, mild-moderate and severe-end stage.

According to univariate analysis, among chronic medical conditions and concomitant therapies, only hepatocellular dysfunction, chronic respiratory failure, heart failure, and NSAID were associated with a higher rate of LA. All intercurrent diseases were significantly associated with LA. Metformin treatment was the only factor which seems to be protective (odds ratio (OR) = 0.68; CI 95%: [0.53–0.86], result not shown).

Neoplasia and insulin were excluded due to *p* value ≥ 0.25. Diuretics were kept in the analysis despite a *p* value = 0.264. Metformin was kept too, despite its apparent protective effect.

#### 3.3.2. Multivariate Analysis

According to our final model (Table 2), among chronic medical conditions, only hepatocellular dysfunction was associated with LA. Surprisingly, early CKD, mild and moderate stage, seemed to have a protective effect. No concomitant therapy was associated with LA occurrence. After adjustment on other risk factors, metformin could not be considered anymore as a protective factor. All the intercurrent diseases that could have been included in the analysis were significantly associated with LA.

Interaction analysis showed that AKI interacted with metformin. We stratified our population on the basis of occurrence of AKI.

Overall, 264 patients presented an AKI, mostly cases (Table 3). There were no differences concerning sex ratio (*p* = 0.993). Proportion of deaths was higher among cases and they were more exposed to metformin than controls but metformin was not significant according to univariate analysis (OR = 1.51; CI 95%: [0.84–2.77]). In multivariate analysis (Table 4), variables significantly associated with LA in patients who presented an AKI were shock, acute respiratory failure, injection of iodinated CM, severe anemia, hepatocellular dysfunction, acute decompensated heart failure, sepsis, and metformin. Early stage CKD (mild and moderate) was considered as a protective factor. According to Hosmer and Lemeshow test, this model fitted well the data (*p* = 0.482). Metformin was a significant risk factor for LA in presence of AKI.

On the other side, 642 patients did not present an AKI. There were more controls than cases (Table 3). There was no significant difference of age (*p* = 0.573) and sex ratio. We observed more deaths in the case group. Controls were treated more often with metformin than cases. In univariate analysis, metformin was a protective factor (OR = 0.48; CI 95% [0.30–0.74]). In multivariate analysis (Table 4), acute respiratory failure, sepsis, acute decompensated heart failure, and hepatocellular dysfunction were significantly associated with occurrence of LA in patients without AKI. Early CKD stage was a protective factor for LA (OR = 0.33; *p* = 0.003). Hosmer and Lemeshow test indicated a good fit (*p* = 0.416). Metformin was no longer associated with LA in type 2 diabetic patients without acute renal dysfunction (OR = 0.86; *p* = 0.628).

#### 3.3.3. Fit of the Final Model

The maximum likelihood *R* ^2^ of our model was 0.878. The predictive accuracy of our model was calculated: sensitivity was 82.78% and specificity was 94.20%. Area under the curve (AUC) is estimated at 0.83.

## 4. Discussion

Several studies on risk factors significantly associated with LA are published but none evaluated the relative importance of these risk factors. This work showed a significant statistical association between a chronic hepatocellular dysfunction, a sepsis, an acute renal, respiratory, or cardiac disease, and occurrence of LA in type 2 diabetic patients.

We performed a backward elimination approach for the multivariable conditional logistic regression model. The method used to choose variables to be included in such model has always been of great concern in epidemiological studies. Backward elimination has the advantage of being objective and its major drawback is the likeliness of real risk factors exclusion. Thus, in addition to likelihood-ratio tests, we made sure at each selection step that the remaining OR did not vary very much. We also informally compared effect sizes between the full model and the final one.

Our results suggested that chronic medical conditions had a minor impact on LA incidence. Hepatocellular dysfunction is the only chronical medical condition significantly associated with LA. This model showed that end or severe stage of CKD (clearance < 30 mL/min) per se is not a risk factor for LA. Early and mild stages of CKD (clearance between 89 and 30 mL/min) even provide protection. This result is constantly found in all the multivariate interaction's analysis.

ACE, ARA, NSAID, diuretics, and iodinated CM have the potential to cause LA indirectly by acute renal failure; ARV drugs by direct action on mitochondrial activity. However they did not appear in the final model. One explanation could be that they have been stopped just before hospitalization.

Regarding metformin, controls were significantly more often treated with metformin (45.70%) than cases (36.75%) (*p* value = 0.015). This can be explained by the fact that contraindications were relatively well respected. In our study, only 5 cases (1.65%) and 1 control (0.16%) with severe and end stage CKD were treated with metformin.

Numerous studies suggested that metformin was safe in patients with moderate renal impairment [20–22]. Since January 2013, metformin is allowed at a dosage of 1.5 g per day in France in case of glomerular filtration rate (GFR) comprised between 30 and 60 mL/min [2]. Guidelines from the NICE published in 2008 suggested that metformin should be reviewed at GFR = 45 mL/min and stopped at 30 [23].

In this study, intercurrent diseases were significantly associated with LA. A study of every LA cause in an English hospital found that most frequent precipitating factors for LA in type 2 diabetic patients were cardiac and respiratory decompensation, AKI, and sepsis [24]. Patients with type 2 diabetes have a 2.5-fold increased risk of AKI, compared to nondiabetic patients [25]. AKI is the most often encountered comorbidity in association with LA [6, 24, 26].

We paid great attention not to include potential confounding factors such as secondary organ insufficiency in the model. When LA was diagnosed lately, we may have included potential outcomes of LA. It can be a potential bias to our study that we try to minimize by the careful study of all medical records.

We identified an interaction between metformin and AKI: metformin was significantly associated with occurrence of LA in case of AKI (OR = 1.79; *p* value = 0.020) but not in patients without AKI. This later result is consistent with what is observed in daily practice or in spontaneous reporting to the pharmacovigilance system and is probably linked to an accumulation of metformin in organism. Injection of iodinated CM is also a potential risk factor in patients with AKI. Thereby, metformin discontinuation before injection of any iodinated CM and biological follow-up of renal function are valuable measures.

The width of confidence intervals for hepatocellular dysfunction, AKI, acute respiratory failure, and sepsis highlighted weak effectives. Indeed, 302 cases could be considered as insufficient to precisely weight risk factors for a rare event. But we were not able to get the data before 2008 because they were not computerized. If such a study should be repeated, it would be in a bigger hospital and within a longer time frame. We chose to match one case to two controls. Matching to more than two controls would have increased power of our study. However we could not match to more than two controls due to low number of patients at extreme ages. A solution could be to realize this study in a larger sample.

A probably useful parameter to consider would have been the advanced stage of diabetes. Micro- and macrovascular complications can lead to more hypoxemia and chronic hyperglycemic and/or hypoinsulinic state to a more important glucose load. Unfortunately information of the disease was often missing in the electronic medical records, since some patients arrived at Grenoble University Hospital for the first time through emergency or intensive care units. Similarly, we could not differentiate the severity of chronic hepatocellular dysfunction based on value of prothrombin ratio.

The mean age of diabetic population in France is 65 [27]. The mean age of our sample is around 69.5. This could be explained by the hospital recruitment: diabetic patients followed up in hospital or requiring a hospitalization may have a more severe condition than those followed up by private practitioners or who do not need hospitalization. Age may influence comorbidities, clinical condition, and the need for more complex care.

Case and control groups were predominantly men; this fact is comparable to the masculine sex ratio described in data from the French National Institute for Public Health Surveillance [27]. There was no sex difference concerning occurrence of LA.

Mortality was much higher among cases (48.3%) than controls (4.3%). Our mortality rate of cases is consistent with previous studies in literature: mortality rate with all biguanides was estimated around 50% [9], even though it was reduced to 26–30% with metformin only [5, 10].

Considering treatment with ACE, ARA, thiazide diuretic, and kidney failure, our population showed similar results compared to ENTRED 2007–2010 [27]. Despite some difference about comorbidities like heart failure, we can consider our sample as a representative one of the French diabetic population.

## 5. Conclusions

According to this study, metformin seemed not to be associated with LA in patients with type 2 diabetes. However, in case of acute renal failure, metformin may be associated with LA. Further studies are needed to precise if metformin could have a deleterious role in other acute medical conditions. But as of now our study allows drawing the hypothesis that metformin withdrawal in case of acute intercurrent disease would probably be as important as checking contraindications before prescribing metformin in order to minimize LA risk.

## Figures and Tables

**Table 1:** Demographic characteristics and comparative analysis of covariates (*N* = 906).

	Cases (*n* = 302)		Controls (*n* = 604)		*p* value
	*n*	(%)	*n*	(%)	
Demographic characteristics					
Age, mean (SD)	69.4 (11.90)	—	69.5 (11.90)	—	0.714
Male	177	(58.6)	354	(58.6)	1
Chronic medical conditions					
CKD	95	(31.5)	224	(37.1)	0.094
Mild-moderate	66	(21.9)	184	(30.5)	0.013
Severe-end stage	29	(9.6)	40	(6.3)	
Hepatocellular dysfunction	47	(15.6)	39	(6.5)	<0.001
Chronic respiratory failure	84	(27.8)	127	(21.0)	0.023
Heart failure	82	(27.2)	94	(15.6)	<0.001
Mitochondrial dysfunction	2	(0.7)	0	(0)	0.111
Neoplasia	38	(12.6)	81	(13.4)	0.728
Pyridoxine deficit	3	(1.0)	13	(2.2)	0.288
Concomitant therapies					
ACE inhibitors	81	(26.8)	186	(30.8)	0.216
ARA	65	(21.5)	155	(25.7)	0.171
Diuretics	150	(49.7)	321	(53.2)	0.323
ARV	1	(0.3)	5	(0.8)	0.670
NSAID	14	(4.6)	12	(2.0)	0.024
Metformin	111	(36.8)	276	(45.7)	0.010
Insulin	129	(42.7)	268	(44.4)	0.636
Iodinated CM	37	(12.3)	17	(2.8)	<0.001
Intercurrent diseases					
Shock	149	(49.3)	8	(1.3)	<0.001
Severe anemia	57	(18.9)	19	(3.2)	<0.001
Dehydration	35	(11.6)	17	(2.8)	<0.001
AKI	184	(60.9)	80	(13.3)	<0.001
Acute hepatic failure	64	(21.2)	12	(2.0)	<0.001
Acute respiratory failure	156	(51.7)	41	(6.8)	<0.001
Myocardial infarction	32	(10.6)	16	(2.7)	<0.001
Acute decompensated heart failure	130	(43.1)	45	(7.5)	<0.001
Sepsis	134	(44.4)	49	(8.1)	<0.001
Convulsions	21	(7.0)	8	(1.3)	<0.001
Intense muscle effort	6	(2.0)	0	—	0.001
Acute artery occlusion	30	(9.9)	28	(4.6)	0.002
Death	146	(48.3)	26	(4.3)	<0.001

**Table 2:** Conditional logistic regression of covariates (*N* = 906).

Variables	Matched OR	CI 95%	*p* value
Chronical medical condition			
No CKD	1	—	—
CKD mild-moderate	0.36	[0.19–0.69]	0.002
CKD severe-end stage	1.62	[0.62–4.25]	0.325
Hepatocellular dysfunction	6.51	[2.78–15.25]	<0.001
Concomitant therapies			
Metformin	1.27	[0.73–2.22]	0.390
Intercurrent diseases			
AKI	9.58	[5.24–17.47]	<0.001
Acute respiratory failure	9.34	[4.76–18.32]	<0.001
Acute decompensated heart failure	3.55	[1.84–6.84]	<0.001
Sepsis	8.28	[4.28–15.99]	<0.001

**Table 3:** Descriptive analysis of patients with (*n* = 264) and without (*n* = 642) AKI.

Variables	Patients with an AKI *n* = 264		Patients without an AKI *n* = 642	
	Cases (%) (*n* = 184)	Controls (%) (*n* = 80)	Cases (%) (*n* = 118)	Controls (%) (*n* = 524)
Demographic characteristics				
Age, mean (SD)	70.34 (11.24)	74.21 (11.55)	68.06 (13.06)	68.75 (11.85)
Male	108 (58.7)	47 (58.8)	49 (41.5)	217 (41.4)
Chronic medical condition				
CKD	66 (35.9)	52 (65.0)	29 (24.6)	172 (32.8)
Mild-moderate	47 (25.5)	42 (52.5)	19 (65.5)	142 (82.6)
Severe-end stage	19 (10.3)	10 (12.5)	10 (34.5)	30 (17.4)
Hepatocellular dysfunction	32 (17.4)	7 (8.8)	15 (12.7)	32 (6.1)
Chronic respiratory failure	45 (24.5)	22 (27.5)	39 (33.1)	105 (20.0)
Heart failure	47 (25.5)	26 (32.5)	35 (29.7)	68 (13.0)
Neoplasia	0	1 (0.5)	21 (17.8)	70 (13.4)
Mitochondrial dysfunction	17 (9.2)	11 (13.8)	1 (0.9)	0
Pyridoxine deficit	2 (1.1)	1 (1.3)	1 (0.9)	12 (2.3)
Concomitant therapies				
ACE inhibitors	50 (27.2)	34 (42.5)	31 (26.3)	152 (29.0)
ARA	45 (24.5)	20 (25.0)	20 (17.0)	135 (25.8)
Diuretics	95 (51.6)	49 (61.3)	55 (46.6)	272 (51.9)
ARV	1 (0.5)	1 (1.3)	0	4 (0.8)
NSAID	11 (6.0)	1 (1.3)	3 (2.5)	11 (2.1)
Metformin	75 (40.8)	25 (31.3)	36 (30.5)	251 (47.9)
Insulin	73 (39.7)	40 (50.0)	56 (47.5)	228 (43.5)
Iodinated CM	20 (10.9)	5 (6.3)	17 (14.4)	12 (2.3)
Intercurrent diseases				
Shock	112 (60.9)	6 (7.5)	37 (31.4)	2 (0.4)
Severe anemia	38 (20.7)	5 (6.3)	19 (16.1)	14 (2.7)
Dehydration	30 (16.3)	10 (12.5)	5 (4.3)	7 (1.3)
Acute hepatic failure	55 (29.9)	3 (3.8)	9 (7.6)	7 (1.7)
Acute respiratory failure	94 (51.1)	12 (15.0)	62 (52.2)	29 (5.5)
Myocardial infarction	21 (11.4)	5 (6.3)	11 (9.3)	11 (2.1)
Acute decompensated heart failure	80 (43.5)	18 (22.5)	50 (42.4)	27 (5.2)
Sepsis	93 (50.5)	13 (16.3)	41 (34.8)	36 (6.9)
Convulsions	10 (5.4)	3 (3.8)	11 (9.3)	5 (1.0)
Intense muscle effort	2 (1.1)	0	4 (3.4)	0
Acute artery occlusion	23 (12.5)	4 (5.0)	7 (5.9)	24 (4.6)
Death	94 (51.1)	10 (12.5)	52 (44.1)	16 (3.1)

**Table 4:** Multivariate analysis with stratification variable: AKI.

Variables	Patients with AKI *n* = 264			Patients without AKI *n* = 642		
	OR	CI 95%	*p* value	OR	CI 95%	*p* value
Chronic medical condition						
CKD mild-moderate	0.42	[0.25–0.73]	0.002	0.33	[0.16–0.68]	0.003
CKD severe-end stage	1.82	[0.80–4.13]	0.149	1.29	[0.49–3.43]	0.607
Hepatocellular dysfunction	5.29	[2.56–10.95]	<0.001	5.17	[2.29–11.67]	<0.001
Concomitant therapies						
Metformin	1.79	[1.09–2.93]	0.020	0.86	[0.48–1.55]	0.628
Iodinated CM	8.58	[3.77–19.52]	<0.001	—	—	—
Intercurrent diseases						
Shock	42.06	[17.91–98.78]	<0.001	—	—	—
Severe anemia	5.83	[2.58–3.17]	<0.001	—	—	—
Acute respiratory failure	11.86	[6.79–20.74]	<0.001	12.38	[6.52–23.50]	<0.001
Acute decompensated heart failure	5.19	[2.88–9.36]	<0.001	6.29	[3.12–12.66]	<0.001
Sepsis	4.34	[2.45–7.70]	<0.001	6.87	[3.61–13.07]	<0.001

## Abbreviations

ACE: Angiotensin converting enzyme
AKI: Acute kidney injury
ARA: Angiotensin II receptor antagonists
ARV: Antiretroviral
CKD: Chronic kidney disease
CM: Contrast media
GFR: Glomerular filtration rate
ICD: International Classification of Diseases
LA: Lactic acidosis
NSAID: Nonsteroidal anti-inflammatory drugs
OR: Odds ratio
ROC: Receiver Operator Characteristic.
